# Age-Related Expression of IFN-λ1 *Versus* IFN-I and Beta-Defensins in the Nasopharynx of SARS-CoV-2-Infected Individuals

**DOI:** 10.3389/fimmu.2021.750279

**Published:** 2021-11-10

**Authors:** Charly Gilbert, Caroline Lefeuvre, Laurence Preisser, Adeline Pivert, Raffaella Soleti, Simon Blanchard, Yves Delneste, Alexandra Ducancelle, Dominique Couez, Pascale Jeannin

**Affiliations:** ^1^ Univ Angers, Université de Nantes, CHU Angers, Inserm, CRCINA, SFR ICAT, Angers, France; ^2^ Laboratory of Immunology and Allergology, Angers University Hospital, Angers, France; ^3^ Laboratory of Virology, Angers University Hospital, Angers, France; ^4^ Univ Angers, CHU Angers, HIFIH, SFR ICAT, Angers, France

**Keywords:** IFN - interferon, defensin, nasopharyngeal mucosa, SARS – CoV – 2, COVID - 19, ageing, mucosal immunity

## Abstract

SARS-CoV-2 coronavirus infection induces heterogeneous symptoms, ranging from asymptomatic to lethal forms. Severe forms usually occur in the elderly and/or individuals with comorbidities. Children generally remain asymptomatic to primary infection, suggesting that they may have an effective local innate immune response. IFN-I and -III have non-redundant protective roles against SARS-CoV-2, although sometimes damaging the host. The expression and role of anti-viral peptides during SARS-CoV-2 infection have thus far been little studied. We aimed to identify the innate immune molecules present at the SARS-CoV-2 entry point. We analyzed the mRNA levels of type I (IFN-α and -β) and type III (IFN-λ1-3) interferons and selected antiviral peptides (*i.e.*, β-defensins 1-3, α-defensins [HNP1-3, HD5] pentraxin-3, surfactant protein D, the cathelicidin LL-37 and interleukin-26) in nasopharyngeal swabs from 226 individuals of various ages, either infected with SARS-CoV-2 (symptomatic or asymptomatic) or negative for the virus. We observed that infection induced selective upregulation of IFN-λ1 expression in pediatric subjects (≤15 years), whereas IFN-α, IFN-β, IFN-λ2/λ3, and β-defensin 1-3 expression was unaffected. Conversely, infection triggered upregulation of IFN-α, IFN-β, IFN-λ2/λ3, and β-defensin 1-3 mRNA expression in adults (15-65 years) and the elderly (≥ 65 years), but without modulation of IFN-λ1. The expression of these innate molecules was not associated with gender or symptoms. Expression of the interferon-stimulated genes IFITM1 and IFITM3 was upregulated in SARS-CoV-2-positive subjects and reached similar levels in the three age groups. Finally, age-related differences in nasopharyngeal innate immunity were also observed in SARS-CoV-2-negative subjects. This study shows that the expression patterns of IFN-I/-III and certain anti-viral molecules in the nasopharyngeal mucosa of SARS-CoV-2-infected subjects differ with age and suggests that susceptibility to SARS-CoV-2 may be related to intrinsic differences in the nature of mucosal anti-viral innate immunity.

## Introduction

Severe acute respiratory syndrome related coronavirus 2 (SARS-CoV-2), a recently emerged enveloped RNA betacoronavirus, is responsible for the current pandemic coronavirus disease 2019 (COVID-19). SARS-CoV-2 is mainly transmitted through respiratory droplets and the nasal and nasopharyngeal mucosa are the preferred sites of viral entry. Epithelial cells in this area constitutively express the SARS-CoV-2 receptor angiotensin-converting enzyme 2 (ACE2) and its associated receptor transmembrane serine protease type II (TMPRSS2) (1–3). The clinical manifestations of COVID-19 infection are very heterogeneous, ranging from asymptomatic to lethal forms, due not only to viral progression in the lower respiratory tract but also to exacerbated inflammatory response. Severe forms of COVID-19 are more likely to occur in males, the elderly, and/or people with comorbidities (4, 5). In contrast, children generally develop asymptomatic or moderate forms, and some studies suggest that this could be partly due to a more effective innate immune response during primary infection compared to adults and elderly (6).

Type-I (IFN-I) and type-III interferons (IFN-III) are natural antiviral mediators. After viral entry into target cells, the recognition of viral nucleic acids by the signaling pattern recognition receptors TLR3, TLR7, TLR9 and RIG-I/MAD-5 triggers the production of IFN-I (IFN-α, IFN-β) and IFN-III (IFN-λ1-3) by various cell types, including epithelial cells (7, 8). Although IFN-I and IFN-III have overlapping properties, they exert unique and non-redundant roles in protecting against viruses (9, 10). IFN-I and IFN-III signal *via* IFNAR and IFNLR, respectively, of which the expression is ubiquitous for IFNAR and restricted to the epithelium for IFNLR (11). Accordingly, it has been proposed that IFN-I induces systemic responses whereas IFN-III-induced responses are restricted to the mucosa (12–14).

IFN-I and IFN-III induce a common signaling pathway involving the ISGF3, leading to the expression of interferon-stimulated genes (ISGs), which participate to the inhibition of viral replication (11). Nevertheless, the panels of ISGs induced by IFN-I and IFN-III and the kinetic of induction are not superimposable (15) and are independent of receptor abundance (16). Moreover, IFN-I favor the initiation of anti-viral adaptive immune responses through the activation of dendritic cells and priming of CD4^+^ and CD8^+^ T-cell responses.

Several studies have emphasized the role of IFN-I and IFN-III in protecting against SARS-CoV-2. Genetic polymorphisms associated with a defective IFN-I production (17) or the induction of anti-IFN-I/IFN-λ3 autoantibodies have been linked to severe forms of COVID-19 (18, 19). Compared to other highly pathogenic coronaviruses and common respiratory RNA viruses, SARS-CoV-2 is a poor inducer of IFN-I response *in vitro* and in animal models (20, 21). The current concept is that a delayed or low IFN-I response fails to control viral replication and favors viral persistence, unabated inflammation, and reduced adaptive immune responses (22).

Aside from IFN-I and IFN-III, the humoral arm of innate immunity also includes antiviral pattern recognition molecules (PRM). Among them, ficolins, collectins such as surfactant proteins A and D (SP-A and SP-D), and pentraxin-3 (PTX3) have been shown to prevent viral entry into cells and/or facilitate the clearance of opsonized viruses (23–25). The cathelicidin LL-37 and interleukin 26 (IL-26) inhibit viral replication thanks to their capacity to bind to viral nucleic acids (26). Some β-defensins (hBD1-3) alter the viral membrane, reducing viral infectivity. They exert a protective role throughout the respiratory tract and are active against many viruses (27, 28). Nevertheless, the expression and roles of PRM during SARS-CoV-2 infection remain poorly described. SP-D have been shown to bind to the envelope protein (29) and the α-defensin 5 (HD5), originally described in Paneth cells, acts as a competitive agonist for the binding of SARS-CoV-2 to ACE2 (30).

The objective of this study was to evaluate the expression of selected innate immunity molecules at the SARS-CoV-2 entry point by comparing the expression of IFN-I, IFN-III and antiviral PRMs, in nasopharyngeal samples from individuals infected or not by SARS-CoV-2, either asymptomatic or with moderate symptoms.

## Results

### SARS-CoV-2 Infection Differentially Upregulates IFN-I and IFN-III mRNA Expression According to Age

We analyzed by RT-qPCR *IFNA*, *IFNB*, *IFNL1*, and *IFNL2L3* mRNA levels in the nasopharyngeal mucosa of subjects infected (n=147) or not (n=79) with SARS-CoV-2. SARS-CoV-2 infection was determined by RT-qPCR and the amount of virus present in the samples estimated using cycle threshold (Ct) values.

**Figure 1:**
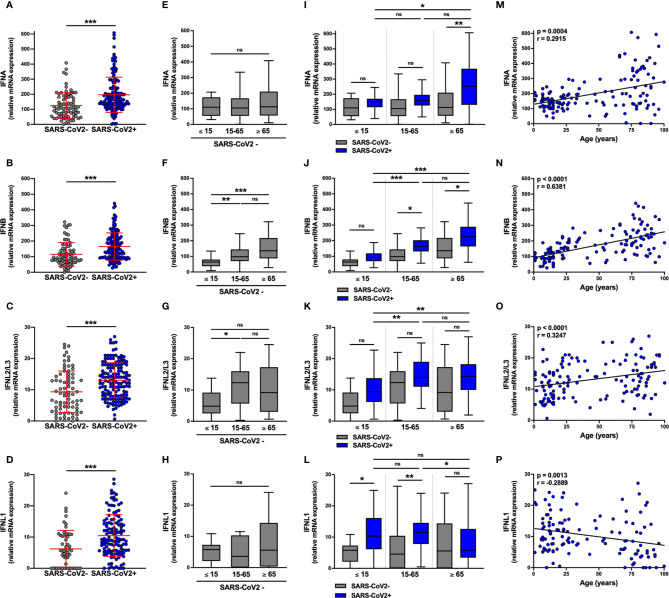
IFN-I and IFN-III transcript levels are differentially increased in SARS-CoV-2-positive patients. The expression of *IFNA*, *IFNB*, *IFNL2/L3*, and *IFNL1* transcripts were determined by RT-qPCR in nasopharyngeal samples from SARS-CoV-2-infected (n=147, blue) or non-infected (n=79, grey) individuals. Data from the two groups are compared as a whole **(A–D)**, or according to the age of the individuals: ≤ 15 years old, 15-65 years old and ≥ 65 years old **(E–L)**. IFN transcript levels and age were correlated **(M–P)**. Each symbol represents a single individual. Boxplots represent the median and 25th to 75th percentiles and whiskers denote the maximum and minimum values. Data are compared using Mann-Whitney **(A–D)**, Kruskal-Wallis test followed by Dunn’s multiple comparison test **(E–L)**, or Spearman’s correlation **(M–P)**. *p < 0.05, **p < 0.005, ***p < 0.001. ns, not significant.

IFN-I (*IFNA* and *IFNB*) mRNA expression (**Figures 1A, B**) was ≈10 fold higher than IFN-III (*IFNL2L3* and *IFNL1*) (**Figures 1C, D**) in non-infected subjects. The levels of these transcripts were equivalent for both sexes (**Figures S1A–D**). Surprisingly, among non-infected individuals, basal levels of *IFNB* mRNA were lower in pediatric (≤ 15 years) than in adult (15-65 years) and elderly (≥ 65 years) subjects and basal levels of *IFNL2L3* mRNA were lower in pediatric (≤ 15 years) than in adult (15-65 years) (**Figures 1F, G**), whereas *IFNA* and *IFNL1* mRNA levels were equivalent, regardless of age (**Figures 1E, H**).

*IFNA*, *IFNB*, *INFL1*, and *IFNL2L3* mRNA levels were significantly higher in SARS-CoV-2-infected subjects than in SARS-CoV-2-negative subjects (**Figures 1A–D**), although there was no difference according to gender (**Figures S1A–D**) or symptoms (**Figure S1E–H**) with equivalent SARS-CoV-2 Ct values in symptomatic and asymptomatic subjects. (**Figures S2A, B**). Normalization of the Ct over housekeeping genes expression led to a similar observation, with no significant difference between symptomatic and asymptomatic patients (**Figure S2D**).

Moreover, the levels of IL-6, TNF-α and IL-8 transcripts were low or undetectable and, when detectable, the levels were not different between SARS-CoV-2-positive and SARS-CoV-2-negative subjects (data not shown), suggesting that differences in the expression of IFN were not associated to the inflammatory response.

We then analyzed the transcript levels in SARS-CoV-2-infected subjects by age group (**Figures 1I–L**); importantly SARS-CoV-2 Ct values were equivalent in the three age groups in both asymptomatic and symptomatic subjects (**Figures S2C, E**). Compared to non-infected subjects, *IFNA* transcript levels were significantly higher in elderly but not adult and pediatric subjects (**Figure 1I**) and *IFNB* transcript levels were elevated in adult and elderly but not in pediatric subjects (**Figure 1J**). Similarly, in support of these observations, there was a positive correlation between IFN-I transcript levels and the age of infected subjects (**Figures 1M, N**).

Concerning IFN-III transcripts, *IFNL1* mRNA levels were significantly higher in SARS-CoV-2-infected pediatric subjects and adults than in elderly subjects (**Figure 1L**), as shown by a negative correlation between *IFNL1* mRNA levels and the age of infected subjects (**Figure 1P**). For *IFNL2/L3*, the infection was associated with a trend towards increased expression in each group, although not significantly (**Figure 1K**), with a positive correlation between transcript levels and the age of infected subjects (**Figure 1O** **)**. In addition, the levels of IFN-III transcripts **(** **Figures S1K, L** **)**, but not those of IFN-I (**Figures S1I, J** **)**, negatively correlated with the SARS-CoV-2 Ct values.

The analysis of ≥65 years individuals presenting or not at least one comorbidity (hypertension, kidney insufficiency, diabetes, or obesity) did not reveal any significant differences in the expression of IFN-I/-III mRNA (data not shown). Moreover, results showed that the relative *IFNA* and *IFNB* mRNA levels are not significantly different between the 3 age groups among uninfected and infected subjects, whether symptomatic or not (data not shown).

**Figure 2:**
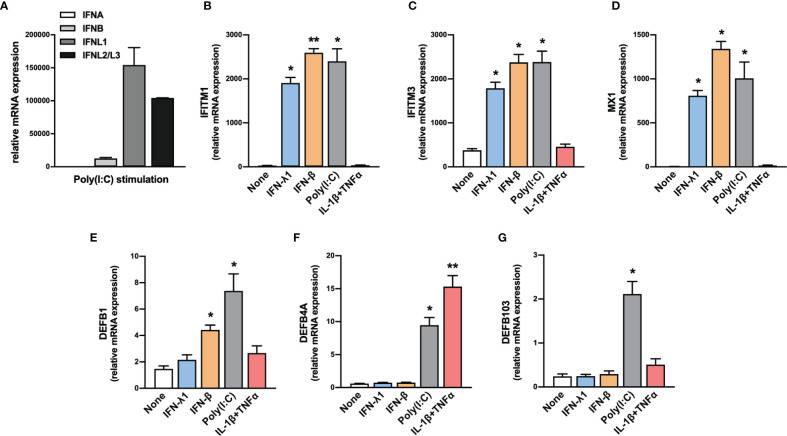
Expression of IFNs, ISGs and β-defensins by HNEpC upon stimulation. **(A)** The expression of *IFNA*, *IFNB*, *IFNL1*, and *IFNL2/L3* transcripts was determined by RT-qPCR in human nasal epithelial cells (HNEpC) following poly(I:C) stimulation (n=3). **(B–G)** The expression of *IFITM1*, *IFITM3*, *MX1*, *DEFB1*, *DEFB4A*, and *DEFB103* transcripts was determined by RT-qPCR in HNEpC following IFN-β, l’IFN-λ1, poly(I:C), or IL-1β + TNFα stimulation for 24h (n=3). Mean ± SEM. Data are compared using one-way ANOVA test followed by Dunnett’s multiple comparisons test **(B–G)**. *p < 0.05, **p < 0.005.

Nasopharyngeal swabs consist predominantly of epithelial cells. Thus, we evaluated the expression of IFN-I and IFN-III transcripts by human primary nasal epithelial cells (HNEpC) in response to poly(I:C), a TLR3 agonist that mimics a viral infection. Results confirmed the expression of IFN-β and IFN-III transcripts by HNEpC in response to poly(I:C) **(** **Figure 2A** **)**.

In conclusion, these results (i) demonstrate an increase in the expression of IFN-I and IFN-III transcripts in SARS-CoV-2-infected subjects and (ii) show that their basal expression and the increase in their expression in infected subjects varies with age. Specifically, only *IFNL1* expression significantly increased in SARS-CoV-2-infected pediatric subjects, whereas *IFNA* transcript levels were lower than those detected in elderly subjects and *IFNB* and *IFNL2/L3* levels were lower than those detected in adult and elderly subjects. On the contrary, the expression of IFN-I mRNA increased in elderly subjects compared with pediatric SARS-CoV2+ subjects, whereas *IFNL1* expression was not modulated.

### SARS-CoV-2 Infection Increases the Expression of IFITM1, IFITM3, and MX1 Transcripts Independently of Age

**Figure 3:**
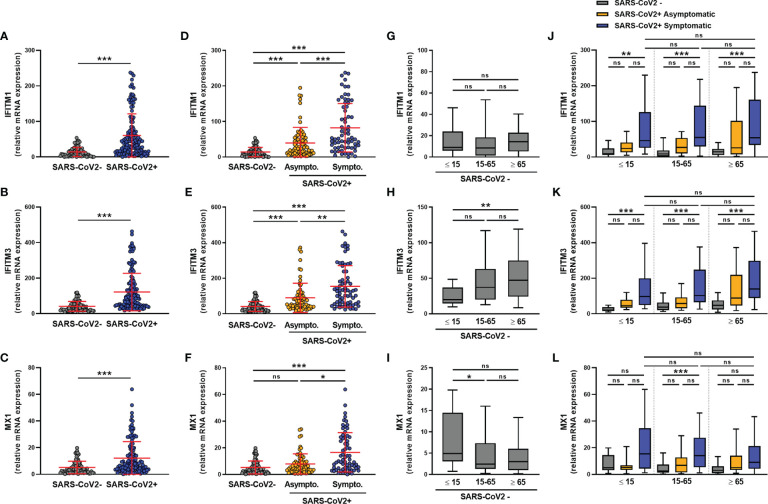
ISG transcript levels are increased in SARS-CoV-2-positive patients. The expression of *IFITM1*, *IFITM3* and *MX1* mRNA was determined by RT-qPCR in nasopharyngeal samples from SARS-CoV-2-infected (n=147) or non-infected (n=79) individuals. A global comparison was performed between data from the two groups **(A–C)**, a comparison was performed between symptomatic (n=72) and asymptomatic (n=75) SARS-CoV-2-infected individuals **(D–F)**, and a comparison was analyzed according to the age of individuals: <=15 years old, 15-65 years old and ≥ 65 years old) **(G–L)**. Each symbol represents a single individual. Boxplots represent the median and 25th to 75th percentiles and whiskers denote the maximum and minimum values. Data are compared using Mann-Whitney **(A–C)** or Kruskal-Wallis test followed by Dunn’s multiple comparison test **(D–L)**. *p < 0.05, **p < 0.005, ***p < 0.001. ns, not significant.

We next assessed the expression of the ISGs *IFITM1*, *IFITM3*, and *MX1*. The expression of these ISGs was higher in SARS-CoV-2-infected than uninfected subjects **(** **Figures 3A–C** **)**, with *IFITM1*, *IFITM3*, and *MX1* mRNA levels being significantly higher in symptomatic than in asymptomatic subjects **(** **Figures 3D–F** **)**. Moreover, in non-infected subjects, *IFITM1* mRNA levels did not vary with age **(** **Figure 3G** **)**, whereas *IFITM3* expression was higher in elderly than pediatric subjects **(** **Figure 3H** **)** and *MX1* expression was higher in pediatric than adult subjects **(** **Figure 3I** **)**. The transcript levels of these transcripts did not vary by sex, whether the subjects were infected or not with SARS-CoV-2 **(** **Figures S3A–C** **)**.

Regardless of age, symptomatic subjects showed higher levels of *IFITM1* and *IFITM3* transcripts than uninfected subjects **(** **Figures 3J, K** **)**. A similar but non-significant trend was observed for the expression of the *MX1* transcript **(** **Figure 3L** **)**.

We observed a negative correlation between the transcript levels of the three transcripts and the SARS-CoV-2 Ct values, suggesting that their expression is associated with the amount of virus present in the nasopharyngeal mucosa, irrespective of the age of the infected subjects **(** **Figures S3D–F** **)**.

In parallel, we tested the ability of HNEpC to express these ISGs in response to various stimuli. We observed an increase in *IFITM1*, *IFITM3*, and *MX1* transcript levels in response to IFN-β, IFN-λ1, and poly(I:C) **(** **Figures 2B–D** **)**. By contrast, the expression of these genes was not induced in response to an inflammatory stimulus (IL-1β+TNFα), confirming their specific induction in response to IFN-I/III **(** **Figures 2B–D** **)**.

In conclusion, *IFITM1*, *IFITM3* and *MX1* expression increased in the nasopharyngeal mucosa of all SARS-CoV-2 infected subjects, regardless of age, and correlated with the SARS-CoV-2 Ct values.

### SARS-CoV-2 Infection Differentially Increases the Expression of Beta-Defensins 1-3 Depending on the Age of the Infected Individuals

**Figure 4:**
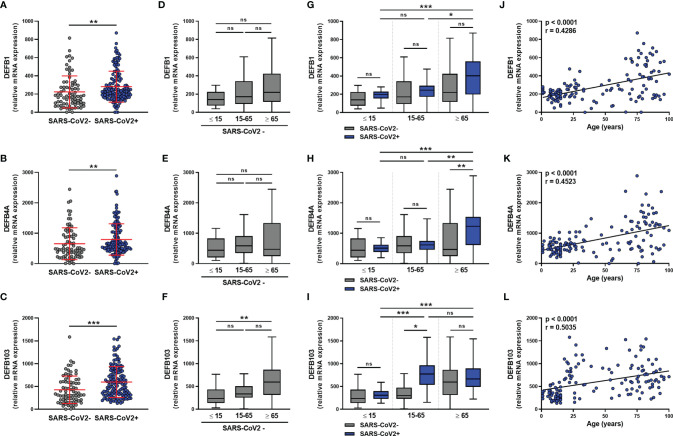
β-defensins transcript levels are differentially increased in SARS-CoV-2-positive patients. hBD1 (*DEFB1*), hBD2 (*DEFB4A*), and hBD3 (*DEFB103*) mRNA levels was determined by RT-qPCR in nasopharyngeal samples from SARS-CoV-2-infected (n=147) or non-infected (n=79) individuals. Data from the two groups are compared as a whole **(A–C)**, or according to the age of individuals: < 15 years old, 15-65 years old and > 65 years old **(D–I)**. hBD1-3 transcript levels and age were correlated **(J–L)**. Each symbol represents a single individual. Boxplots represent the median and 25th to 75th percentiles and the whiskers denote the maximum and minimum values. Data are compared using Mann-Whitney **(A–C)**, Kruskal-Wallis test followed by Dunn’s multiple comparison test **(D–I)**, or Spearman’s correlation **(J–L)**.*p < 0.05, **p < 0.005, ***p < 0.001. ns, not significant.

We next analyzed the expression of β-defensins in the nasopharyngeal mucosa of SARS-CoV-2-infected and uninfected subjects. For *DEFB1* (hBD1), *DEFB4A* (hBD2), and *DEFB103* (hBD3) transcripts, results showed a significant increase in the level of all three hBD1-3 transcripts in SARS-CoV-2 infection **(** **Figures 4A–C** **)**, which did not differ according to sex **(** **Figures S4A–C** **)** or between symptomatic and asymptomatic subjects **(** **Figures S4D–F** **)**. hBD1-3 transcripts were detected in subjects not infected with SARS-CoV-2 **(** **Figures 4D–F** **)** and *DEFB103* mRNA was more highly expressed in elderly than pediatric subjects **(** **Figure 4F** **)**.

The analysis of these transcripts in SARS-CoV-2-infected subjects according to age showed that the infection does not upregulate hBD1-3 expression in young subjects **(** **Figures 4G–I** **)**. On the contrary, infection was associated with a trend towards an increase in *DEFB1* and *DEFB103* transcript levels in adults, as well as *DEFB1* and *DEFB4A* transcript levels in elderly subjects **(** **Figures 4G–I** **)**. There was a significant correlation between the levels of the hDB1-3 transcripts and the age of the SARS-CoV-2-infected subjects **(** **Figures 4J–L** **)**.

The analysis of ≥65 years individuals presenting or not at least one comorbidity (hypertension, kidney insufficiency, diabetes, or obesity) did not reveal any significant differences in the expression of hBD1-3 mRNA (data not shown).

In parallel, HNEpC cells expressed *DEFB1*, *DEFB4A*, and *DEFB103* mRNA **(** **Figures 2E–G** **)** and different stimuli controlled hBD1-3 expression. *DEFB1* transcript levels increased in response to IFN-β and poly(I:C) **(** **Figure 2E** **)**, whereas those of *DEFB4A* increased in response to poly(I:C) and IL-1β + TNF-α **(** **Figure 2F** **)**. Only exposure to poly(I:C) resulted in an increase in *DEFB103* transcript levels **(** **Figure 2G** **)**.

The other anti-viral PRMs analyzed, such as human neutrophils peptides (HNP)1-3, human α-defensin 5 (DEFA5), pentraxin 3 (PTX3), surfactant protein D (SP-D), and the amphipathic molecules LL-37 and IL-26, were not detected in nasopharyngeal swabs from infected subjects (data not shown). As expected, the transcripts encoding these PRMs were not detected in HNEpC under basal conditions or in response to various stimuli (data not shown).

In conclusion, our results show that (i) hBD1-3 transcripts were detectable in SARS-CoV-2-infected and uninfected subjects, (ii) the mechanisms of induction of these defensins *in vitro* differ, and (iii) hBD1-3 levels were significantly higher in elderly (hBD1-3) infected subjects than infected pediatric subjects. Finally, SARS-CoV-2 infection did not appear to induce the expression of these β-defensins in young subjects.

**Figure 5:**
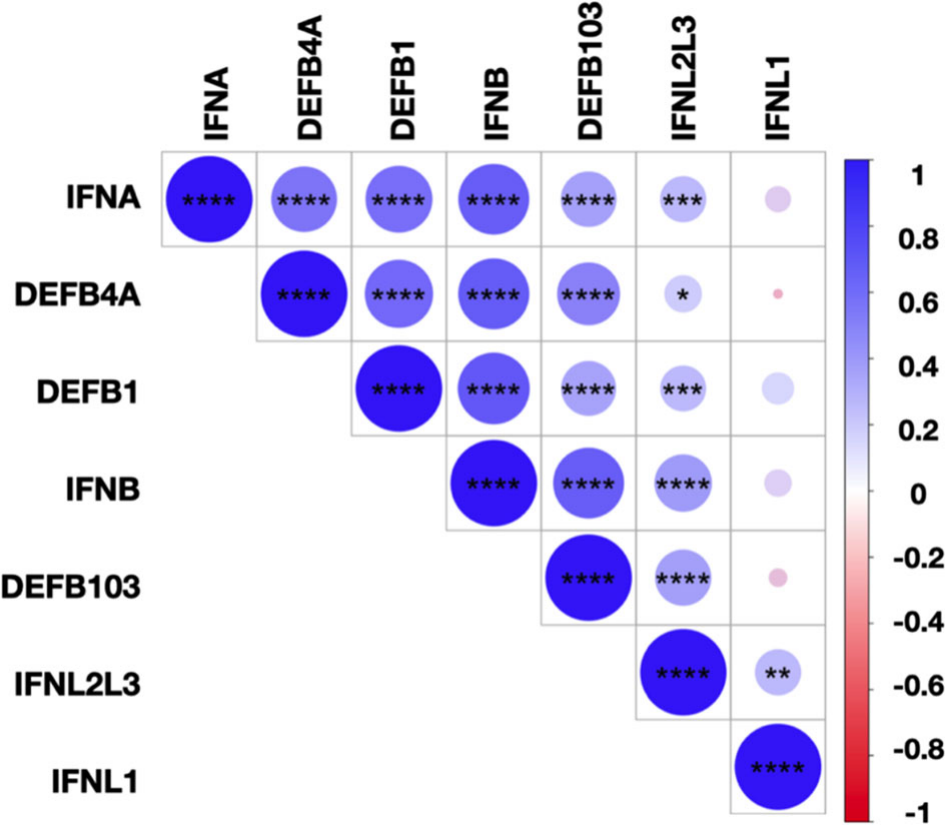
Correlations between IFN-I/-III and hBDs transcript levels. Correlation matrix between IFN-I (*IFNA*, *IFNB*), IFN-III (*IFNL1*, *IFNL2/L3*), and hBD1-3 (*DEFB1*, *DEFB4A*, *DEFB103*) transcripts levels in SARS-CoV-2-infected subjects. The levels of correlation between the transcripts are ordered by hierarchical clustering. The scale represents the correlation coefficient between the different transcripts. The significance of the correlations was calculated using Spearman’s correlation test. *p < 0.05; **p < 0.01; ***p < 0.001; ****p < 0.0001.

Finally, we assessed the correlations between the levels of IFN-I/-III, *DEFB1, DEFB4A*, and *DEFB103* transcripts for all SARS-CoV-2 infected subjects **(** **Figure 5** **)**. We observed close correlations between the transcript levels of IFN-α, IFN-β, IFN-λ2/-λ3, and hBD1-3, suggesting common regulation of these transcripts. *IFNL1* transcript levels correlated with those of *IFNL2/L3* but not IFN-I or hBD1-3, illustrating non-redundant regulation of the expression of these molecules. Importantly, the levels of IL-6, TNF-α and IL-8 transcripts were undetectable or low and, when detectable, not different between SARS-CoV-2-positive and SARS-CoV-2-negative subjects (data not shown), suggesting that differences in the expression of IFN and hBD were not strictly associated to the inflammatory response.

## Discussion

In this study, we show that the expression profiles of IFN-I, IFN-III, and β-defensins in the nasopharyngeal mucosa of SARS-CoV-2-infected subjects differ according to their age rather than sex or the presence of symptoms. The expression of IFN-I transcripts was higher in adult and elderly SARS-CoV-2-infected subjects than pediatric subjects. In the pediatric subjects, only the level of the IFN-λ1 transcript appeared to significantly increase upon infection with SARS-CoV-2.

Type I and III interferons play a protective role against COVID-19. Pretreatment with IFN-I and/or IFN-III reduces the susceptibility of intestinal (31) and lung cells to SARS-CoV-2 infection (32). By contrast, ruxolitinib, which inhibits IFN-induced signaling and depletion of IFNAR and IFNLR receptors, increases infection (32). Highly pathogenic coronaviruses, such as SARS-CoV-2, delay and limit the induction of IFNs (33, 34). The anti-SARS-CoV-2 roles of IFN-I depends on their induction kinetics and level of expression: an early response would be protective, whereas a late and intense response would interfere with development of the adaptive immune response and promote inflammation (35). IFN-β administration is protective in a murine model of SARS-CoV infection if administered early but impairs viral clearance and worsens the pathology if administered late (36).

The higher expression of IFN-λ1 and relatively lower levels of IFN-α and IFN-β at the point of viral entry in pediatric subjects infected with SARS-CoV-2 relative to that of adult and elderly subjects could contribute to explain why pediatric subjects are less prone to severe forms of the disease (37). IFN-III, which are more rapidly produced than IFN-I by nasal epithelial cells in response to various respiratory viruses (10), allow the control of respiratory virus infection at the epithelial barrier, while minimizing the inflammatory response (10). Unlike IFN-I, which are inflammatory due to signaling *via* IRF1 (38), IFN-III, which exert their activities primarily at epithelial barriers, inhibit neutrophil recruitment and function (13, 39) and do not induce IRF1 activation (38). The absence of a pro-inflammatory effect of IFN-III is one of the main arguments in favor of their therapeutic use over that with IFN-I (10, 38, 40). For example, only IFN-λ induces upper respiratory tract protection in a mouse model of Influenza type A virus infection, whereas the antiviral activities of IFN-α and IFN-λ overlap in the lower respiratory tract (41). In an IL-28RA^-/-^ hamster model, IFN-III reduce the spread of SARS-CoV-2, whereas IFN-I exacerbate bronchopneumonia (42). Finally, low serum IFN-λ2 levels are associated with increased severity of COVID-19 in patients infected with SARS-CoV-2 (43).

We compared the ability of infected subjects to respond to IFNs by measuring the transcript levels of the ISGs IFITM1, IFITM3, and MX1. IFITMs inhibit the entry of SARS-CoV-2, SARS-CoV and MERS-CoV into cells (44–46) and MX1 inhibits the viral ribonucleoprotein complex (47–49). A decrease in MX1 expression with age in SARS-CoV-2-infected subjects is associated with an increased risk of severe forms (50). We show that ISG levels do not differ with age in SARS-CoV-2-infected subjects, despite different IFN-I/III transcript levels.

This absence of a difference in expression could be explained by different mechanisms of IFN-I/III feedback and a rapid and transient effect of IFN-I relative to that of IFN-III (15, 31). The results showing that the expression of these ISGs was higher in symptomatic than asymptomatic subjects is consistent with those of a study showing high expression of ISGs in bronchoalveolar lavage of severe SARS-CoV-2-infected subjects (51). Finally, a positive correlation between ISG transcript levels and SARS-CoV-2 Ct values shows the establishment of a graded local response of the nasopharyngeal mucosa, adjusted to the amount of virus, in both young and elderly subjects.

Our results suggest that type-III IFN may be protective against SARS-CoV-2. In agreement with this hypothesis, accumulating evidence suggest that type-III IFNs rather than type-I IFNs are the predominant antiviral cytokines at mucosal barriers (12, 52) and that type-III IFN are more effective than type-I IFN in preventing viral infection, the latter being associated which systemic inflammation and tissue damage (10, 12). Moreover, IFN-λ1 inhibits SARS-CoV-2 replication *in vitro* (31, 53–55). Interestingly, in agreement with a previous study reporting that a delayed type I IFN response promotes exacerbated inflammation (56), Sposito B et al. recently reported that the expression of IFN-λ1 and IFN-λ3 in the upper airways characterizes patients with a mild disease while, in contrast, critically ill patients exhibit a preferential expression of type IFN-I (57). Based on these studies, it was tempting to speculate that administration of IFN-Λ (presumably IFN-Λ1 or IFN-Λ3), at an early stage of COVID-19, would induce a protective antiviral response. In agreement with this hypothesis, recent results from a phase 2 clinical study reported that IFN-Λ1 accelerated viral clearance in outpatients with COVID-19 (58).

The role of antiviral PRMs and, in particular hBDs, is beginning to be studied in SARS-CoV-2 infection. Peptides derived from the murine orthologue of hBD2, as well as hBD-2, which binds to the SARS-CoV-2 spike protein, protect against SARS-CoV-2 infection (59, 60). Here, we show that SARS-CoV-2 infection significantly increases the expression of hBD1-3 transcripts at the viral entry point, with differences in expression depending on the age of the subjects. We observed a positive correlation between IFN-I, IFN-λ2/λ3, and β-defensin levels. Thus, the expression of these beta-defensins did not increase in young subjects infected with SARS-CoV-2, who have higher levels of IFN-λ1. Consistent with these observations, we show that poly(I:C) strongly increases the expression of these three defensins by human nasal epithelial cells and that IFN-I induce/increase the expression of hBD1, whereas IFN-λ1 does not modulate their expression. An increase in the expression of hBD2 and hBD3 has already been reported in certain viral infections (61). Finally, an inflammatory stimulus (IL-1β + TNFα) essentially induces hBD2 expression, showing that the mechanisms of induction of β-defensins are not superimposable or redundant.

We observed that the expression of soluble mediators of innate immunity varies with age, whether or not subjects are infected with SARS-CoV-2. Age-related differences in the innate immune response have been described in the literature. For example, immunosenescence is associated with less effective epithelial barriers, with thin, permeable mucosa and low-grade inflammation (smoldering), which may favor the recruitment of inflammatory cells, such as IFN-I producing plasmacytoid dendritic cells. Sun et al. also reported severe interstitial pneumonia and cytokine storm in aged hACE2 mice (62); similar results were obtained using a mouse-adapted strain of SARS-CoV-2 (63). In addition, the adaptive immune response, which is less effective in primary infections, can lead to an intense and long-lasting compensatory immune response, which can cause tissue damage. On the contrary, the epithelial barrier and lymphoid tissues associated with the mucous membranes (adenoids) in young subjects provide optimal protection and lower expression of anti-viral molecules appears to be sufficient to ensure mucosal protection. Finally, at the molecular level, the expression and function of viral sensors (TLR3, TLR7, TLR8, RIG-I) can vary with age. This also helps to explain the differences in the innate humoral response to the same virus with age (64–66). Thus, in accordance with our observations, the production of soluble innate immune molecules in response to viral sensors is markedly reduced in young subjects (67).

In conclusion, we show that age influences the expression patterns of type-I/III interferons and hBDs induced at the SARS-CoV-2 entry point. Children respond to infection with increased IFN-λ1 expression. On the contrary, adults and the elderly respond to infection by overexpression of IFN-I and hBD1-3. As IFN-λ1 is associated with effective protection of the mucous membranes of the upper respiratory tract in the absence of inflammation, these differences may help to explain why children remain less prone to severe/critical forms of COVID-19. Due to the design of this study, we do not have information on the outcome of SARS-CoV-2 positive subjects. A prospective study will allow evaluating the predictive potential of the cytokine signature on the evolution of the infection in young versus adult subjects.

## Material and Methods

### Specimens and Characteristics of Individuals

This study included 226 residual samples from SARS-CoV-2 diagnosis by quantitative reverse transcriptase – polymerase chain reaction (RT-qPCR) from nasopharyngeal swabs specimens collected and processed by the laboratory of virology of the Angers University Hospital (Angers, France). Individuals were divided into three age groups: pediatric (≤15 years), adult (15-65 years), and elderly (≥ 65 years); and according to the SARS-CoV-2 infection status: symptomatic or asymptomatic individuals with RT-qPCR positive for SARS-CoV-2 infection and individuals with a negative RT-qPCR and no symptoms (**Table 1**). According to the WHO classification, SARS-CoV-2-positive individuals had mild to moderate forms (68). None of the subjects included in this study received antiviral medication at the time of nasopharyngeal sampling. Comorbidities of individuals (hypertension, diabetes, kidney insufficiency or obesity) were collected in clinical records when available. Considering the entire cohort demographic, including age, the sex ratio, and the rate of comorbidities, we found no statistical difference between each group, with the exception of SARS-CoV-2 positive patients which were significantly younger than SARS-CoV-2 negative patients in the 15-65 years group. This study was performed according to the recommendations of the ethics committee of the University Hospital of Angers (agreement 2021–56).

**Table 1:** Clinical data.

		SARS-CoV2-	SARS-CoV2+ Asymptomatic	SARS-CoV2+ Symptomatic	p-value
**≤15 years (n = 72)**					
Age	Median (IQ)	10 (9/14.5)	11 (7.5/13)	10 (3/14)	*: 0.62
Sex	N (M/F)	23 (17/6)	27 (16/11)	22 (12/10)	#: 0.37
Comorbidities					
Hypertension	Nb of individuals (%)	0 (0)	0 (0)	0 (0)	/
Diabetes	Nb of individuals (%)	0 (0)	0 (0)	0 (0)	/
Kidney insufficiency	Nb of individuals (%)	0 (0)	0 (0)	0 (0)	/
Obesity	Nb of individuals (%)	0 (0)	0 (0)	0 (0)	/
**15-65 years (n = 73)**					
Age	Median (IQ)	47 (29/53)	26.5 (23/44)	29 (23/43,5)	*: 0.03
Sex	N (M/F)	30 (15/15)	20 (13/7)	23 (11/12)	#: 0.47
Comorbidities					
Hypertension	Nb of individuals (%)	4 (13%)	1 (5%)	1 (4%)	#: 0.15
Diabetes	Nb of individuals (%)	2 (7%)	1 (5%)	0 (0)	#: 0.47
Kidney insufficiency	Nb of individuals (%)	1 (3%)	1 (5%)	0 (0)	#: 0.59
Obesity	Nb of individuals (%)	1 (3%)	1 (5%)	0 (0)	#: 0.59
**≥65 years (n = 81)**					
Age	Median (IQ)	73.5 (70/84)	82 (76/89)	79 (75.5/85,5)	*: 0.12
Sex	n (M/F)	26 (11/15)	28 (12/16)	27 (12/15)	#: 0.99
Comorbidities					
Hypertension	Nb of individuals (%)	14 (54%)	17 (60%)	14 (52%)	#: 0.79
Diabetes	Nb of individuals (%)	4 (15%)	7 (25%)	10 (37%)	#: 0.20
Kidney insufficiency	Nb of individuals (%)	1 (4%)	5 (18%)	6 (22%)	#: 0.15
Obesity	Nb of individuals (%)	1 (4%)	0 (0)	3 (11%)	#: 0.16

Age, sex, and comorbidities (hypertension, diabetes, kidney insufficiency, obesity) of subjects for whom nasopharyngeal samples were collected are presented according to age group (≤15 years, 15-65 years, ≥ 65 years) as well as SARS-CoV-2 status (SARS-CoV-2 negative, asymptomatic SARS-CoV-2 positive, symptomatic SARS-CoV-2 positive). IQ, interquartile range; M, male; F, female; ^#^data were compared using a Chi-square test; *data were compared using a Kruskal-Wallis test.

### Cell Culture

Human nasal epithelial cells (HNEpC) were purchased from Promocell (Heidelberg, Germany) and cultured in Airway Epithelial Cell Growth medium according to the manufacturer recommendations. In some experiments, cells were seeded at 10^5^ cells/well in 24-well culture plate and rested overnight for attachment. Cells were starved for 6 h in RPMI 1640 medium before a 24 h stimulation with 50 ng/ml IFN-β or IFN-λ1 (both from Peprotech, Cranbury, NJ), 5 µg/ml low molecular weight Poly(I:C) (ranging from 0.2 to 1 kb) (Invivogen, San Diego, CA) or 10 ng/ml IL-1β (Miltenyi Biotec, Bergisch Gladbach, Germany) and 10 ng/ml TNFα (Immunotools, Friesoythe, Germany). Cells were then collected for RNA extraction.

### Determination of SARS-CoV-2 Infection

Nasopharyngeal swabs were placed on viral transport medium and 200 μL of nasopharyngeal sample was extracted on the NucliSens^®^ easyMAG automated platform (Biomérieux, Marcy l’Etoile, France) according to the manufacturer recommendations. Determination of SARS-CoV-2 infection by RT-qPCR was performed on ABI 7500 FAST Real-Time PCR System (Applied Biosystems, Foster city, CA, USA) using primers (nCoV_IP2 and nCoV_IP4) targeting two regions on the RNA-dependent RNA polymerase (RdRP) gene (National Reference Center of respiratory viruses; Institut Pasteur, Paris, France).

### Quantification of IFN-I/-III, ISG and β-Defensins 1-3 mRNA

Total RNA from HNEpC was extracted using the RNeasy^®^ micro kit from Qiagen (Hilden, Germany). RNA extracted from nasopharyngeal samples and HNEpC were reverse-transcribed with the SuperScript™ First-Strand II Synthesis System (Invitrogen, Waltham, MA, USA) using random hexamers (Themo Fisher Scientific, Carlsbad, CA, USA) on the Biometra TOne Thermocycler (Analytik Jena AG, Jena, Germany). The quantification of *IFNA, IFNB, IFNL1, IFNL2/L3, IFITM1, IFITM3, MX1, DEFB1, DEFB4A* and *DEFB103* mRNA by qPCR was performed on a LC480 (Roche Diagnostic) using SYBR^®^ Green I Master mix (Thermo Fischer Scientific). Relative quantification was performed using the method developed by Vandesompele et al. (69), using RPS18 and EF1A as references. The calculation method is based on the conversion of the linear Cq values into a logarithmic scale using the efficiency of PCR as an exponential function. The geometric mean of selected housekeeping genes is used as a normalization factor, allowing eliminating inter-sample variations. The value 1 is given to the calibrator with the highest expression level within a series; the levels of expression of the other genes are then calculated compared to the calibrator. Primer sequences are listed in the **Supplementary Table S1**.

### Statistical Analysis

Clinical data presented in **Table 1** were compared using Chi-Square test for qualitative variables, or Kruskal-Wallis for quantitative variables. For the cohort analysis, the comparison of mRNA levels and SARS-CoV-2 Ct values was realized using nonparametric Mann-Whitney test for two-group comparison or Kruskal-Wallis test followed by Dunn’s multiple comparisons test when more than two groups were compared. For multiple comparisons, the family-wise error rate was corrected using Bonferroni correction. Correlations were determined using Spearman’s correlation. Atypical values in each group were identified and removed using Tukey’s fences. For the HNEpC experiments, data were analyzed by one-way ANOVA with Welch’s correction, followed by Dunnett’s multiple comparisons tests. Results were considered statistically significant for p-values < 0.05. Statistical analyses were performed with Prism V8.0 (GraphPad Software Inc, La Jolla, CA) and R software (version 4.0.2). Correlation matrix were generated using the “corrplot” package in R software.

## Data Availability Statement

The raw data supporting the conclusions of this article will be made available by the authors, without undue reservation.

## Ethics Statement

The studies involving human participants were reviewed and approved by Ethics committee of the University Hospital of Angers (agreement 2021–56). Written informed consent from the participants’ legal guardian/next of kin was not required to participate in this study in accordance with the national legislation and the institutional requirements.

## Author Contributions

CG designed the experiments, performed the experiments, analyzed, and interpreted the data, and wrote the manuscript. LP, RS, and SB performed the experiments, acquired the data, and contributed to the analyze of the data. CL and AP provided clinical samples, collected and analyzed the data. YD and AD contributed to design the study and discussed the data. DC and PJ contributed to the overall study design, supervised the project, and revised the manuscript. All authors contributed to the article and approved the submitted version.

## Funding

This work was supported by institutional grants from the French National Institute of Health and Medical Research (INSERM) and the University of Angers and by a grant from CSL Behring. Funders had no role in the design of this study, analysis and interpretation of the data and decision to submit.

## Conflict of Interest

The authors declare that the research was conducted in the absence of any commercial or financial relationships that could be construed as a potential conflict of interest.

## Publisher’s Note

All claims expressed in this article are solely those of the authors and do not necessarily represent those of their affiliated organizations, or those of the publisher, the editors and the reviewers. Any product that may be evaluated in this article, or claim that may be made by its manufacturer, is not guaranteed or endorsed by the publisher.
