# Supplementary material for: Age-Related Expression of IFN-λ1 Versus IFN-I and Beta-Defensins in the Nasopharynx of SARS-CoV-2-Infected Individuals
Source: Front Immunol. 2021 Nov 10;12:750279. doi: 10.3389/fimmu.2021.750279 (PMC8631500; doi:10.3389/fimmu.2021.750279)
Supplement: Supplementary file 1 [file DataSheet_1.pdf]

Supplemental Material

Table S1. List and sequence of primers used in this manuscript for qPCR amplification

Gene		Sequence (5'- > 3')
RPS 18	F	CCTTTGCCATCACTGCCA
	R	GTTCCACCTCATCCTCAGTG
EF1a	F	TGATATGGTTCCTGGCAAGC
	R	TAGCCTTCTGAGCTTTCTGG
IFNA	F	ATCTACGATGGCCTCGCC
	R	GTGCCAGGAGCATCAAGG
IFNB	F	TCTCCACTACAGCTCTTTCC
	R	CTCATAGATGGTCAATGCGG
IFNL1	F	CTGCACCACATCCTCTCC
	R	TTGACGTTCTCAGACACAGG
IFNL2/L3	F	TCGCTTCTGCTGAAGGACTGCA
	R	CCTCCAGAACCTTCAGCGTCAG
MXA	F	CCGTGACGGATATGGTCCGGC
	R	CTGGAAGTGGAGGCGGATCAGC
IFITM1	F	TGCTGTCTGGGCTTCATAGC
	R	GGTAGACTGTCACAGAGCCG
IFITM3	F	CCCTCTTCATGAACCCCTGC
	R	CCTGGAAGATCAGCACTGGG
DEFB1	F	GGTAACTTTCTCACAGGCCTTGG
	R	TCCCTCTG TAACAGGTGCCTTG
DEFB103	F	TTATTGCAGAGTCAGAGGCGGC
	R	CTTTCTTCGGCAGCATTTTCGGC
DEFB4A	F	ATAGGCGATCCTGTTACCTGCC
	R	CATCAGCCACAGCAGCTTCTTG

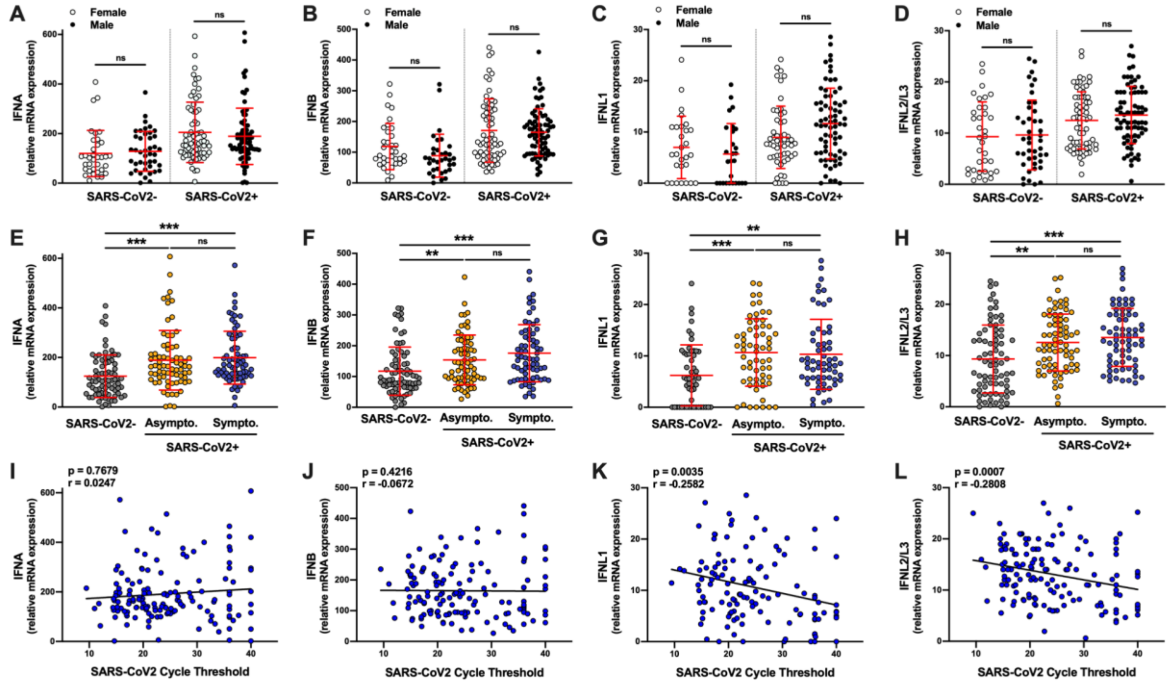

Figure S1. IFN-I et -III gene expression. The levels of mRNA for IFNA, IFNB, IFNL1 and IFNL2/L3 were analyzed by RT-qPCR in nasopharyngeal samples from individuals infected (n=147) or not (n=79) by SARS-CoV-2. Results are compared by female (white) or male (black) gender (**A-D**) or by the presence of symptoms (**E-H**). SARS-CoV2 Ct values of infected subjects are shown as a function of the expression levels of IFN-I and -III transcripts (Spearman rank correlation test) (**I-L**). Data were compared Kruskal-Wallis test followed by Dunn's multiple comparison test (**A-H**), or Spearman's correlation (**I-L**) *p < 0,05; ** p< 0.01; ***p<0.001; ns = not significant.

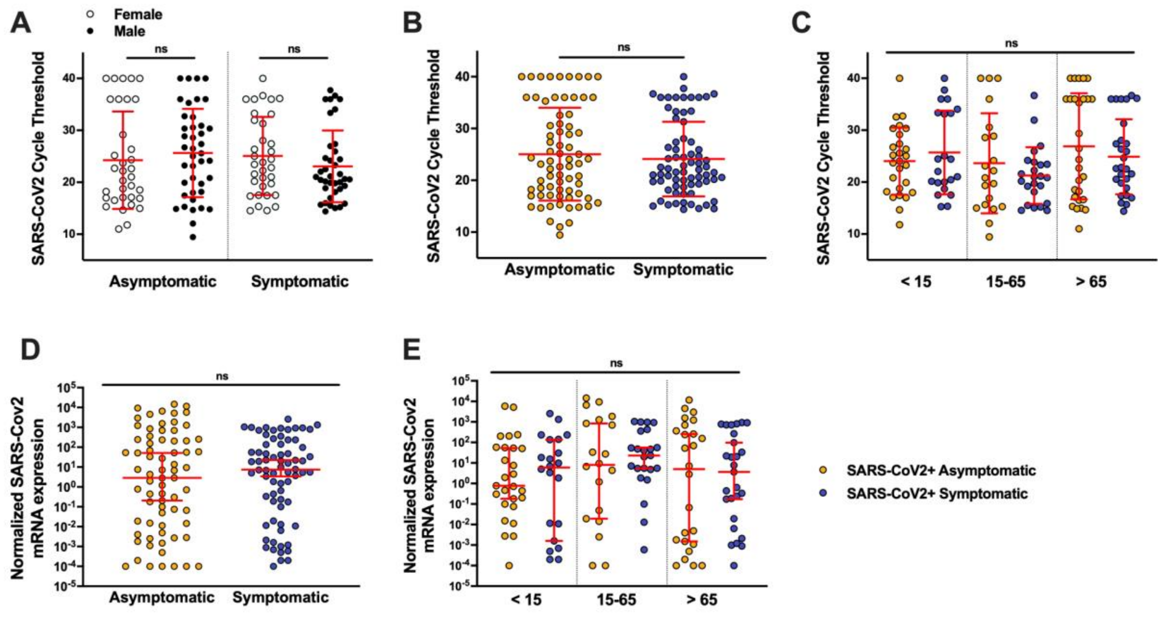

Figure S2. SARS-CoV-2 Ct value. SARS-CoV2 Ct values are compared by female (white) or male (black) gender (**A**), presence of symptoms in all subjects (**B**), or by age groups (**C**). The expression of SARS-CoV-2 mRNA normalized over the expression of housekeeping genes are compared depending on the presence of symptoms in all subjects (**D**), or by age groups (**E**). Each symbol represents a single individual. Data were compared using Mann-Whitney (**B&E**) or Kruskal-Wallis test followed by Dunn's multiple comparison test (**A,C,E**).*p < 0,05; ** p< 0.01; ***p<0.001; ns = not significant.

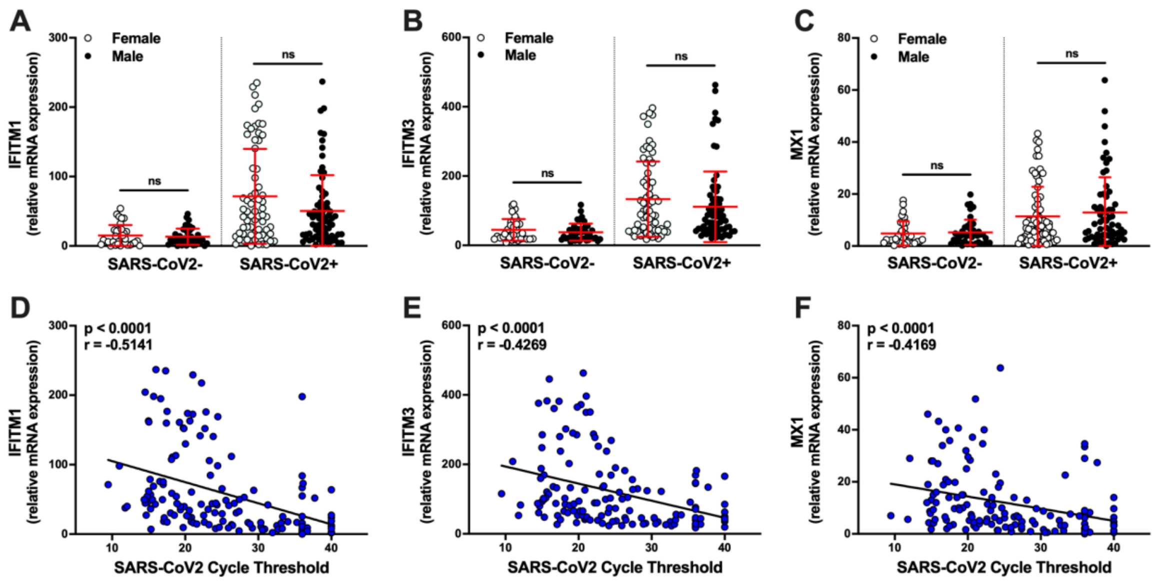

Figure S3. IFITM1, IFITM3 and MX1 gene expression. The levels of mRNA for IFITM1, IFITM3 and MX1 were analyzed by RT-qPCR in nasopharyngeal samples from individuals infected (n=147) or not (n=79) by SARS-CoV-2. Results are compared by female (white) or male (black) gender (**A-C**). SARS-CoV2 Ct values of infected subjects are shown as a function of the expression levels of IFITM1, IFITM3 et MX1 transcripts (Spearman rank correlation test) (**D-F**). Each symbol represents a single individual. Data were compared using Kruskal-Wallis test followed by Dunn's multiple comparison test (**A-C**), or Spearman's correlation (**D-F**). *p < 0,05; ** p< 0.01; ***p<0.001; ns = not significant.

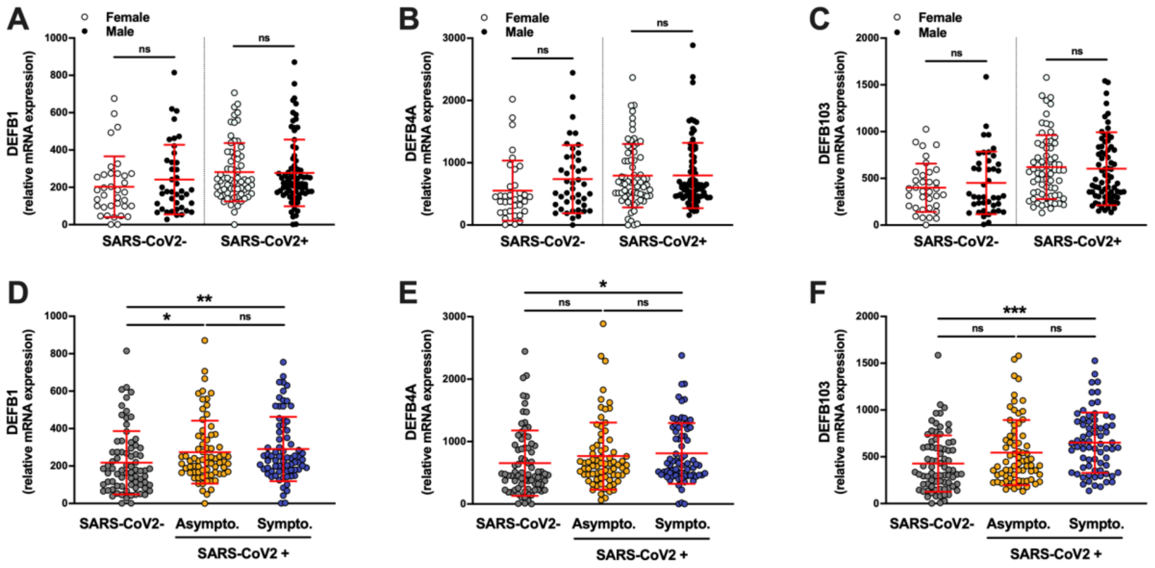

Figure S4. β-defensins (hBD1-3) gene expression. The levels of mRNA for hBD1 (*DEFB1*), hBD2 (*DEFB4A*) et hBD3 (*DEFB103*) were analyzed by RT-qPCR in nasopharyngeal samples from individuals infected (n=147) or not (n=79) by SARS-CoV-2. Results are compared by female (white) or male (black) gender (**A-C**) or by the presence of symptoms (**D-F**). Each symbol represents a single individual. Data were compared using Kruskal-Wallis test followed by Dunn's multiple comparison test (**A-F**). *p < 0,05; ** p< 0.01; ***p<0.001; ns = not significant.
